# Functionality-packed additively manufactured porous titanium implants

**DOI:** 10.1016/j.mtbio.2020.100060

**Published:** 2020-06-03

**Authors:** I.A.J. van Hengel, F.S.A. Gelderman, S. Athanasiadis, M. Minneboo, H. Weinans, A.C. Fluit, B.C.J. van der Eerden, L.E. Fratila-Apachitei, I. Apachitei, A.A. Zadpoor

**Affiliations:** a Additive Manufacturing Laboratory, Department of Biomechanical Engineering, Faculty of Mechanical, Maritime, and Materials Engineering, Delft University of Technology, the Netherlands; b Department of Orthopedics, University Medical Center Utrecht, Utrecht, the Netherlands; c Department of Medical Microbiology, University Medical Center Utrecht, Utrecht, the Netherlands; d Department of Internal Medicine, Erasmus Medical Center, Rotterdam, the Netherlands

**Keywords:** Multifunctional surfaces, Biofunctionalization, Antimicrobial implant, Additive manufacturing, Strontium, Silver nanoparticles

## Abstract

The holy grail of orthopedic implant design is to ward off both aseptic and septic loosening for long enough that the implant outlives the patient. Questing this holy grail is feasible only if orthopedic biomaterials possess a long list of functionalities that enable them to discharge the onerous task of permanently replacing the native bone tissue. Here, we present a rationally designed and additive manufacturing (AM) topologically ordered porous metallic biomaterial that is made from Ti-6Al-4V using selective laser melting and packs most (if not all) of the required functionalities into a single implant. In addition to presenting a fully interconnected porous structure and form-freedom that enables realization of patient-specific implants, the biomaterials developed here were biofunctionalized using plasma electrolytic oxidation to locally release both osteogenic (i.e. strontium) and antibacterial (i.e. silver ions) agents. The same single-step biofunctionalization process also incorporated hydroxyapatite into the surface of the implants. Our measurements verified the continued release of both types of active agents up to 28 days. Assessment of the antibacterial activity *in vitro* and in an *ex vivo* murine model demonstrated extraordinarily high levels of bactericidal effects against a highly virulent and multidrug-resistant *Staphylococcus aureus* strain (i.e. USA300) with total eradication of both planktonic and adherent bacteria. This strong antibacterial behavior was combined with a significantly enhanced osteogenic behavior, as evidenced by significantly higher levels of alkaline phosphatase (ALP) activity compared with non-biofunctionalized implants. Finally, we discovered synergistic antibacterial behavior between strontium and silver ions, meaning that 4–32 folds lower concentrations of silver ions were required to achieve growth inhibition and total killing of bacteria. The functionality-packed biomaterial presented here demonstrates a unique combination of functionalities that make it an advanced prototype of future orthopedic biomaterials where implants will outlive patients.

## Highlights

• Rationally designed and selective laser melted porous titanium bone implants.
• Surface biofunctionalization by plasma electrolytic oxidation with strontium and silver nanoparticles.
• Biofunctionalized implants eradicated methicillin-resistant *Staphylococcus aureus* *in vitro* and *ex vivo* within 24 h.
• Novel synergistic antibacterial behavior between strontium and silver ions.
• Enhanced osteogenic differentiation of preosteoblastic cells after 11 days.

## Graphical abstract

**Figure:**
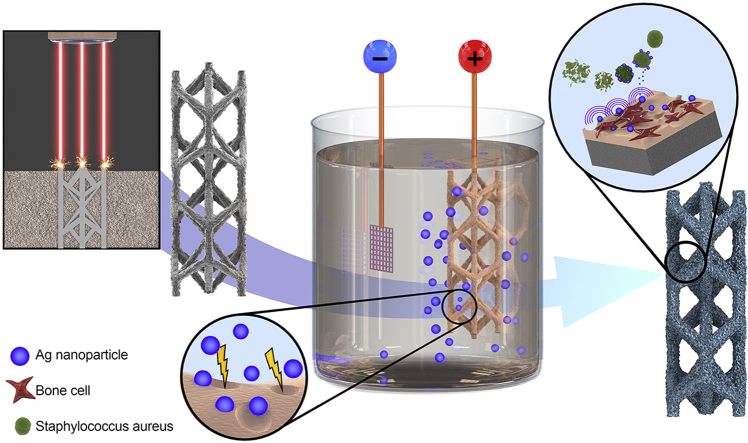

## 1 Introduction

Orthopedic implants are the jewels of the medical device industry: they help keep tens of millions of people mobile. Similar to all other functional devices, however, they too have a limited service life. Generally, loosening marks the end of the lifespan of orthopedic implants when debilitating pain sets in and the patient's mobility diminishes to the point of complete evanescence.

Implant loosening can generally be categorized as being either aseptic or septic. The holy grail of orthopedic implant design is to ward off both aseptic and septic loosening for long enough that the implant outlives the patient. Researchers have been questing for this holy grail using a host of methodological approaches such as the synthesis of new biomaterials [1], the surface biofunctionalization of implants [2,3], conceiving implants with bone-mimicking mechanical properties [[4], [5], [6], [7]], and the local delivery of active agents [8,9].

Frequently, however, these developments fall short of the ultimate goal, as the strenuous task of permanently replacing biological tissues requires mustering more than one single craft. Therefore, multiple functionalities need to be packed into one single piece of implant. To prevent aseptic loosening for as long as possible, one should improve the primary stability of the implant [10,11], minimize stress shielding through bone-mimicking mechanical properties [[12], [13], [14], [15]], provide a fully interconnected volume-porous structure to allow for optimal bony ingrowth [16,17], and stimulate the osteogenic differentiation of stem cells [18,19]. As for septic loosening, both short-term and long-term implant-associated infections (IAIs) should be staved off through obliteration of the bacteria reaching the implant surface post-operatively, hematogenously, or contiguously [20]. This lengthy list of design objectives necessitates a reinterpretation of the term ‘multifunctional biomaterials’ as biomaterials that are packed with many multidomain functionalities that have been traditionally considered difficult to obtain and at times even contradictory.

A number of recent developments in additive manufacturing (AM) technologies [[21], [22], [23], [24], [25]], rational design processes [26,27], and surface biofunctionalization techniques [[28], [29], [30]] have, however, made it feasible to incorporate many or all of the aforementioned functionalities into one single piece of orthopedic implant. Here, we present an advanced prototype of such functionality-packed biomaterials that has the potential of meeting most (if not all) of our design objectives. First, we used rational design principles and AM for fabrication of topologically ordered porous titanium that present a fully interconnected porous microarchitecture to allow for optimal bony ingrowth [21,22,31], while exhibiting highly adjustable bone-mimicking mechanical properties [[32], [33], [34]] that minimize stress shielding. We also used the form-freedom offered by AM [35] to create bespoke implants that maximize their primary stability. In the case of the present study, the bespoke geometry is that of the murine femora used for our *ex vivo* animal experiments. These miniaturized geometries also demonstrate the potential of our approach for fabrication of implants with fine geometrical details. These three functionalities are not the only advantages of our complex topological design: it was also optimized to increase the surface area of our implants by more than threefold as compared with a corresponding solid implant [36]. This multifold increase in the surface area amplifies the effects of the unique surface biofunctionalization technique used for addressing the remaining design objectives.

In addition to being functionality-packed, much of the novelty of the biomaterials presented here originates from the surface biofunctionalization technique applied to simultaneously prevent aseptic loosening through stimulation of the osteogenic differentiation of stem cells, as well as septic loosening through both short- and long-term delivery of antibacterial agents from the entire volume of the AM porous biomaterials. Although the osteogenic [[37], [38], [39], [40]] and antibacterial [29,[41], [42], [43]] properties of the locally delivered active agents (i.e. strontium and silver nanoparticles, respectively) are known, we explored the use of both agents simultaneously to generate multifunctional properties on the complex geometry of our highly porous AM implants.

## 2 Materials and methods

### 2.1 Topological design and AM

We used a hexagonal unit cell with an ultrahigh surface-to-volume ratio [36] to design the microarchitecture of our topologically ordered porous structures. Miniaturized implants with a geometry optimized for implantation in murine femora were designed with a length of 4 cm and a diameter of 0.5 mm, resulting in a 35.6 surface-to-volume ratio. The specimens were AM using a customized selective laser melting (SLM) equipment (SLM-125, Realizer, Borchem, Germany) at the Additive Manufacturing Laboratory (TU Delft, Delft, The Netherlands) using a YLM-400-AC Ytterbium fiber laser (IPG Photonics Corporation, Oxford, United States) operated inside an argon atmosphere with less than 0.2% oxygen content. Medical-grade (grade 23, ELI) Ti-6AL-4V powder (AP&C, Boisbriand, Quebec, Canada) with a spherical morphology, particles sizes between 10 and 45 μm, and a layer thickness of 50 μm was used. Laser processing was performed with an exposure time of 300 μs, a wavelength of 1070 ± 10 nm, and a laser power of 96 W, resulting in a laser spot size of 145 μm. After SLM manufacturing, the loose powder particles were removed by vacuum cleaning. The specimens were subsequently ultrasonicated in acetone followed by immersion in 96% ethanol and demineralized water for 5 min each.

### 2.2 Surface biofunctionalization

The surface of AM porous implants was biofunctionalized using plasma electrolytic oxidation (PEO) in a custom-made setup consisting of an AC power source (50 Hz, type ACS 1500, ET Power Systems Ltd, Eyam, United Kingdom), a data acquisition board (SCXI, National Instruments, Austin, Texas, United States), a computer interface, and a double-walled glass electrolytic cell containing 800 ml electrolyte [44,45]. The PEO electrolyte contained 0.15 M calcium acetate, 0.02 M calcium glycerophosphate, 0.3 M strontium acetate, and 3.0 g/L silver nanoparticles (AgNPs) (Sigma-Aldrich, St. Louis, Missouri, United States). AgNPs with a spherical morphology and a size distribution of 7–25 nm were dispersed in the PEO electrolyte by ultrasonication of 2 times 3 min to obtain a homogenous suspension in the electrolyte. In between the sonication steps, the electrolyte was stirred for 5 min at 500 rpm with a magnetic stirrer (IKA-Werke GmBH & Co. KG, Staufen, Germany) and stir bar of 40 × 8 mm (VWR, Radnor, Pennsylvania, United States).

PEO processing was performed under galvanostatic conditions with a current density of 20 A/dm^2^. The implant served as the anode whereas a stainless-steel cylinder placed against the inner wall of the electrolytic cell formed the cathode. To maintain the homogeneity of the electrolyte, it was continuously stirred at 500 rpm. Furthermore, the temperature of the electrolyte was kept in a range of 6 ± 2 °C through a thermostatic bath (Thermo Haake, Karlsruhe, Germany) connected to the electrolytic cell. During the PEO process, the voltage-time (V-t) transients were recorded at a sampling rate of 1 Hz. After surface biofunctionalization, the implants were rinsed in running tap water for 1 min and sterilized by heat treatment for 1 h at 110 °C in an oven (Nabertherm GmbH, Lilienthal, Germany).

As-manufactured implants without any surface biofunctionalization were designated as the non-treated (NT) group. Additional experimental groups included PEO-treated implants without strontium or silver (PT), as well as those with strontium (PT-Sr), AgNPs (PT-Ag), or both (PT-AgSr).

### 2.3 Biomaterial characterization

#### 2.3.1 Scanning electron microscopy

The surface morphology of the specimens was studied using a scanning electron microscopy (SEM) (JSM-IT100LA, JEOL, Tokyo, Japan) with electron beam energies in the range of 5–20 kV and a working distance of 10 mm. Before imaging, the implants (*n* = 3/group) were coated with a gold layer of 5 ± 2 nm to enhance electrical conductivity. To analyze the chemical composition of the implant surface, energy-dispersive X-ray spectroscopy (EDS) was performed.

#### 2.3.2 X-ray diffraction

The phase compositions of the specimens from the NT, PT, and PT-Sr groups were studied with a D8 advanced diffractometer (Bruker, Billerica, Massachusetts, United States) with Bragg-Brentano geometry and Lynxeye position sensitive detectors. The following settings were applied: CuKα radiation detector = LL 0.11 W 0.14, divergence slit = V6, scatter screen height = 5 mm, current = 40 mA, and voltage = 45 kV. No sample spinning was applied during the experiments. The specimens were measured using a coupled θ - 2θ scan from 20 to 120°, a step size of 0.034° 2θ, and a counting speed of 10 s/step. The obtained data were analyzed using the DiffracSuite.Eva (version 4.1) software (Bruker).

#### 2.3.3 Inductively coupled plasma optical emission spectroscopy

The release kinetics of strontium and silver ions were analyzed using inductively coupled plasma optical emission spectroscopy (ICP-OES). Biofunctionalized specimens (*n* = 3 per experimental group, length = 1.5 cm) were submerged in 1 ml phosphate buffered saline (PBS) in a brown glass vial and kept at 37 °C in a water bath. The medium was sampled after 0.5, 1, 2, 4, 7, 14, and 28 days to measure the concentrations of silver and strontium ions using a spectrometer (Spectro Arcos, Kleve, Germany).

### 2.4 Antibacterial assays

#### 2.4.1 Preparation bacterial inoculum

To prepare a bacterial inoculum, a single colony of methicillin-resistant *Staphylococcus aureus* (MRSA) (strain = USA300 [[46], [47], [48]]) was suspended in either 3 ml tryptic soy broth (TSB) or cation-adjusted Mueller Hinton (CAMH) broth and incubated for 2 h at 37 °C while shaking at 120 rpm. After incubation, the optical density at 600 nm (OD_600_) was measured, and the required bacterial inoculum was prepared based on the OD_600_ value. The prepared inoculum was quantified by plating 10 μl triplicates of 10-fold serial dilutions on blood agar plates (Becton Dickinson, Franklin Lakes, United States) followed by overnight incubation at 37 °C and quantification of colony forming units (CFUs).

#### 2.4.2 Minimal inhibitory concentration and minimal bactericidal concentration

The minimal inhibitory concentration (MIC) and minimal bactericidal concentration (MBC) of Ag^+^ and Sr^2+^ ions, as well as combinations thereof were determined in CAMH broth using silver nitrate and strontium acetate (both from Sigma-Aldrich, St. Louis, United States). An MRSA USA300 inoculum of OD_600_ 0.09 was prepared of which 65 μl was transferred to 10 ml of CAMH broth. Two-fold serial dilutions were prepared in a 96-well plate starting from 2 mM for Ag^+^ and 80 mM for Sr^2+^. Subsequently, 50 μl of bacterial inoculum and 50 μl of both Ag^+^ and Sr^2+^ dilutions were added together in a 96-well plate and incubated overnight at 37 °C under static conditions. The following day, the MIC was scored as the lowest concentration of Ag^+^ and Sr^2+^ where no turbidities were present. To determine the MBC, 10 μl aliquots of each well were plated on blood agar plates and incubated overnight at 37 °C, followed by CFU counting. The MBC was noted as the lowest concentration of Ag^+^ and Sr^2+^ where no colonies were observed.

#### 2.4.3 Leachable antibacterial assay

To determine the antibacterial leaching activity, agar plates were prepared from Luria broth consisting of 200 g tryptone, 100 g yeast powder, 240 g Agar No.1 (all from Oxoid, ThermoFisher Scientific, Massachusetts, United States), and 200 g NaCl dissolved in 20 L ultrapure water. A bacterial inoculum of OD_600_ 0.01 was prepared in TSB, and bacterial suspensions were evenly distributed over the surface of the agar plates using a sterile swab. Subsequently, 1.5 cm implants were placed on the agar surface and incubated at 37 °C in a humid environment for 24 h. After incubation, the area of the zone of inhibition was measured with image processing software (Photoshop CS6, Adobe, California, United States) to determine the antibacterial leaching activity (*n* = 3 per group).

#### 2.4.4 Quantitative bactericidal assay

To quantify the bactericidal activity, the numbers of adherent and non-adherent (i.e. planktonic) CFU were quantified. Therefore, 4 implants of 1 cm were inserted in 200 μl MicroAmp® Fast Reaction Tubes (Life Technologies, Carlsbad, California, United States) with a bacterial inoculum of 2 × 10^3^ CFU MRSA USA300 in 100 μl TSB + 1% glucose and incubated overnight at 37 °C under static conditions (*n* = 3 per group). To determine the number of adherent CFU, the implants were washed 3 times in PBS, ultrasonicated for 3 min in 200 μl PBS, and 10 μl aliquots of 10-fold serial dilutions were plated on blood agar plates. The number of non-adherent CFU were quantified from the inoculation medium by plating 10 μl aliquots of 10-fold dilutions on blood agar plates. Following overnight incubation at 37 °C, the number of CFU were quantified.

#### 2.4.5 Biofilm formation and characterization

To evaluate the formation of biofilms, implants (*n* = 2 per group) were statically incubated at 37 °C in 1 ml TSB + 1% glucose and inoculated with 10^8^ CFU/ml MRSA USA300. After 48 h, the implants were washed with PBS and fixated in McDowels fixative (4% paraformaldehyde and 1% glutaraldehyde in 10 mM phosphate buffer at pH 7.4). Biofilm formation was analyzed by dehydrating the fixated implants as per the following procedure: rinsing in demineralized water for 5 min and dehydration in 50% ethanol for 15 min, 70% ethanol for 20 min, 96% ethanol for 20 min, and hexamethyldisilazane for 15 min. Subsequently, the implants were dried in air for 2 h and coated with a gold layer of 5 ± 2 nm.

#### 2.4.6 Ex vivo animal experiments

To assess the intraosseous antibacterial properties, the biofunctionalized implants were evaluated *ex vivo* in murine femora explanted from mouse cadavers by the Central Laboratory Animal Institute (Utrecht University). After removal of the surrounding tissue, the femora were sterilized in 70% ethanol for 10 min and subsequently submerged in demineralized water for 10 min. A hole of 0.5 mm was drilled through the epicondyle into the intramedullary canal and bone marrow was removed with a syringe. To simulate *in vivo* conditions, 2 μl of PBS was inserted into the medullary cavity. Before implantation, the implants were inoculated with an inoculum of 200 CFU MRSA USA300 in 2 μl PBS, left to dry in air for 15 min, and implanted into the femur.

As a control for the ethanol sterilization of the femora, one femur did not receive an implant (negative control). To validate the model, 2 μl of tetracycline (50 mg/ml, Sigma-Aldrich, St.Louis, Missouri, United States) was injected into the bone cavity after implantation with an inoculated NT implant. After implantation, the femora were incubated in 0.5 ml tubes at 37 °C on a rotating platform to simulate intraosseous fluid flow. After 24 h, the femora were submersed in 800 μl PBS with 15 zirconia beads (Ø 2 mm, BioSpec, Bartlesville, Oklahoma, United States) and homogenized using a MagNA Lyser (Roche Diagnostics, Risch-Rotkreuz, Switzerland) at 7000 rpm for 2 cycles of 30 s and cooled on ice in between. From the resulting homogenate, 10-fold serial dilutions were prepared on blood agar plates, incubated overnight at 37 °C, and the numbers of CFU were quantified.

### 2.5 Osteogenic cell assays

#### 2.5.1 Cell seeding and culturing

Osteoblastic murine MC3T3-E1cells (Sigma-Aldrich) were cultured for 7 days in culture medium consisting of α minimum essential medium, supplemented with 1% penicillin-streptomycin and 10% fetal bovine serum (all from ThermoFisher Scientific). The medium was refreshed every 2–3 days. Before cell seeding, the implants were cut to 1 cm length and sterilized at 110 °C for 1 h in an oven (Nabertherm GmbH, Lilienthal, Germany). Cell seeding was performed by inserting an implant in a 0.2 ml tube with 1.5 × 10^5^ MC3T3-E1 cells in 100 μl culture medium. Subsequently, the implants were incubated at 37 °C and 5% CO_2_ in a horizontal position and tilted every 20 min for 2 h in total. After seeding, the implants were placed in a 48-well plate with 200 μl fresh medium. After 2 days of culturing, osteogenic differentiation was induced by the addition of 50 μg/ml ascorbic acid and 4 mM β-glycerophosphate (all from Sigma-Aldrich). Thereafter, the medium was refreshed every 2–3 days. Two independent experiments were performed (each time in quadruplicates).

#### 2.5.2 Presto blue assay

The metabolic activity of the MC3T3-E1 cells was determined by a PrestoBlue assay (Thermofisher, Waltham, MA, United States) after 1, 3, 7, and 11 days of culture. The same replicates were used for all time points. The implants (*n* = 4 per group) were incubated in 200 μl fresh culture medium supplemented by 20 μl PrestoBlue cell viability reagent for 1 h at 37 °C. Thereafter, the fluorescence was measured at an excitation wavelength of 530 nm and an emission wavelength of 595 nm with a Victor X3 microplate reader (PerkinElmer, Nederland B.V., Groningen, The Netherlands). Furthermore, we determined the cell seeding efficiency on the implants (*n* = 4 per group) immediately after seeding by deducting the number of live cells present in the culture medium from the total number of seeded cells.

#### 2.5.3 Alkaline phosphatase assay

The alkaline phosphatase (ALP) activity of the differentiated MC3T3-E1 cells was determined 11 days after cell seeding. The implants (*n* = 4 per group) were rinsed with PBS and 250 μl PBS-Triton added (8% NaCl, 0.2% KCl, 1.44% Na_2_HPO_4_, 0.24% KH_2_PO_4_ and 0.1% Triton X-100 in H_2_O). The cells were dissociated from the implants by ultrasonication for 10 min and incubated with 100 μl *p*-nitrophenyl phosphate (pNPP, Sigma-Aldrich) at 37 °C for 10 min. Subsequently, 250 μl NaOH was added to stop the reaction. The absorbance was then measured at a wavelength of 405 nm with the same Victor X3 microplate reader. To determine the ALP activity, a standard curve was prepared by addition of 100 μl PBS-Triton and 250 μl NaOH to each well and the total protein content was determined with a BCA protein assay kit (Invitrogen). Subsequently, the ALP levels were normalized to the total protein content.

#### 2.5.3 Cell morphology

The number and morphology of MC3T3-E1 cells on the surface of the implants were assessed by SEM after 5 days of incubation. The implants were fixed in McDowels fixative (4% paraformaldehyde and 1% glutaraldehyde in 10 mM phosphate buffer at pH 7.4) and stored at 4 °C. Before SEM imaging, the implants were rinsed twice in demineralized water for 5 min and dehydrated in ethanol (15 min in 50%, 20 min in 70% and 20 min in 96%). Subsequently, the implants were dried in air for 2 h, coated with a gold layer of 5 ± 2 nm, and analyzed by SEM (*n* = 2 per group).

### 2.6 Statistical analysis

All data are presented as mean ± standard deviation. Statistical analysis was performed with GraphPad Prism (GraphPad Software, La Jolla, California, United States) using one-way and repeated-measured ANOVA tests. The differences between various experimental groups were considered as statistically significant when *p < 0.05*.

## 3 Results

### 3.1 Surface morphology and PEO biofunctionalization

**Fig. 1:**
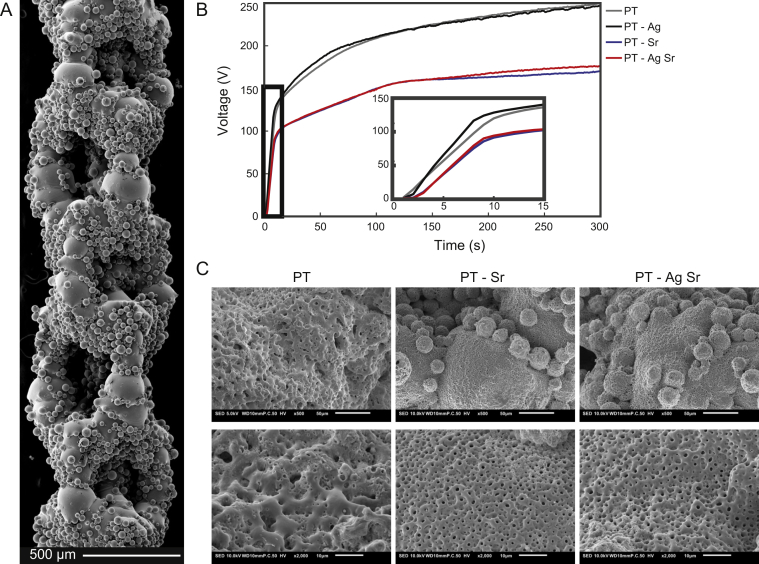
(A) SEM micrograph demonstrating the surface morphology of the implants after SLM. (B) The V-t transients recorded during the PEO processing of the SLM implants with different electrolytes. (C) Low (500×) and high (2000×) magnification SEM images of the PT, PT-Sr and PT-AgSr implants after 300 s of oxidation. SLM, selective laser melting; PEO, plasma electrolytic oxidation; SEM, scanning electron microscopy.

AM porous biomaterial presented highly porous structures with various partially molten Ti-6Al-4V powder particles attached to the surfaces (Fig. 1A). The V-t curves (Fig. 1B) recorded for the specimens from the PT and PT-Ag groups demonstrated similar transients, whereas those of the PT-Sr and PT-AgSr groups had a much lower final voltage. Up until dielectric breakdown, the voltage increased with 14 ± 1 V/s for the PT and PT-Ag implants after which the slope of the curve reduced to 0.49 V/s and plasma discharging started at 115 ± 5 V, resulting in a final voltage of 249 ± 6 V. For the PT-Sr and PT-AgSr implants, the voltage rose slower with a rate of 11.3 ± 1 V/s. Furthermore, the increase rate of the voltage was lower (i.e. 0.28 V/s) as compared with the specimens from the PT and PT-Ag groups resulting in final voltages of 170 ± 4 for the PT-Sr and PT-AgSr groups. SEM analysis demonstrated uniform coverage of the implant surfaces with a micro-/nanoporous oxide layer (Fig. 1C). The addition of strontium acetate in the PEO electrolyte resulted in smaller pore sizes for the PT-Sr group as compared to the PT group. The addition of AgNPs did not alter the surface morphology of the biofunctionalized implants compared with PT and PT-Sr implants.

### 3.2 Surface chemistry and phase composition of biofunctionalized implants

**Fig. 2:**
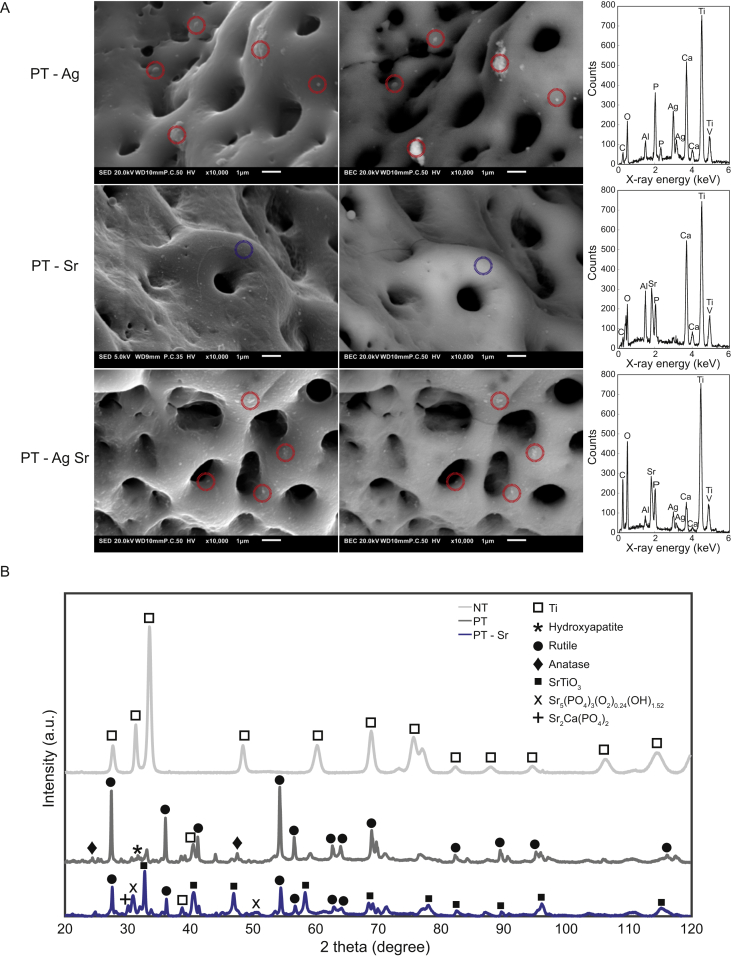
(A) Secondary (left) and backscattered (right) SEM images demonstrating the location and chemical composition of silver nanoparticles (red dotted circles) and the TiO_2_ matrix (blue dotted circles) on PT-Ag, PT-Sr and PT-AgSr implant surfaces. (B) The XRD spectra of NT, PT, and PT-Sr specimens. SEM, scanning electron microscopy; XRD, X-ray diffraction.

Spot EDS measurements demonstrated the presence of Ca, P, Ti, Al, and V on the surface of all biofunctionalized specimens (Fig. 2A). Sr was detected on the surface of the specimens from the PT-Sr and PT-AgSr groups. Furthermore, backscattered SEM images, as well as EDS measurements verified the presence of AgNP on the surface of PT-Ag and PT-AgSr implants. AgNPs were spread homogenously over the surface and fully embedded in the TiO_2_ layer. Phase analysis with X-ray diffraction (XRD) demonstrated a crystalline TiO_2_ layer consisting of mainly rutile, as well as lesser extents of anatase phases (Fig. 2B). Moreover, the hydroxyapatite phase (Ca_10_(PO_4_)_5.64_(CO_3_)_0.66_(OH)_3.03_) and strontium apatite (Sr_5_(PO_4_)_3_(O_2_)_0.24_(OH)_1.52_) were detected on PT and PT-Sr specimens, respectively. In addition, strontium titanium oxide (SrTiO_3_) and strontium-Ca/P (Sr_2_Ca(PO_4_)_2_) were observed on the surface of the PT-Sr implants.

### 3.3 Ion release and antibacterial activity

#### 3.3.1 Ion release kinetics

**Fig. 3:**
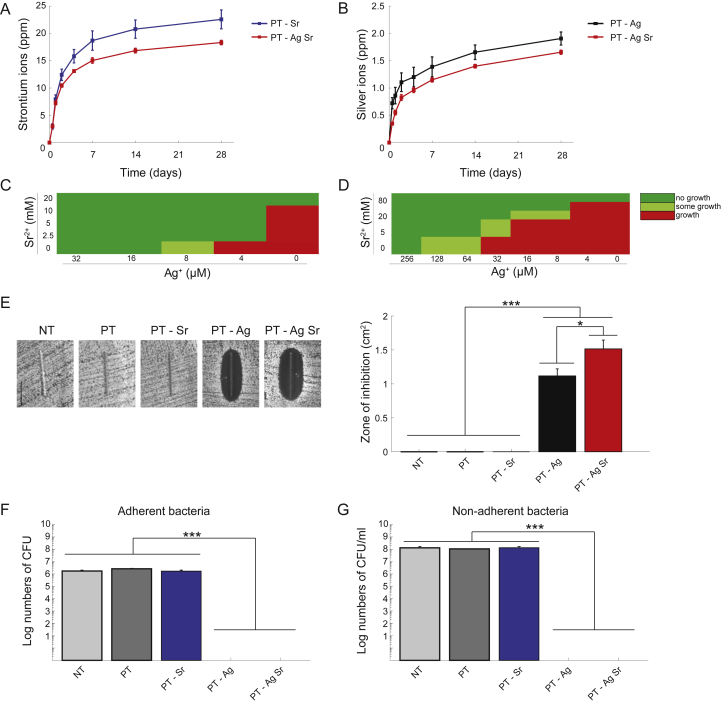
The ion release profile and *in vitro* antibacterial activity against MRSA USA300. The cumulative ion release of (A) Sr^2+^ and (B) Ag^+^ ions released from biofunctionalized implants in PBS as determined by ICP-OES. (C) The results of the minimum inhibitory and (D) bactericidal concentration tests demonstrating the level of bacterial growth for different concentrations of Ag^+^ and/or Sr^2+^ ions. (E) The photographs (left) and size (right) of the inhibition zones formed around the specimens after 24 h of incubation on an agar plate with an inoculum of 10^7^ CFU/ml. (F) The number of adherent and (G) non-adherent bacteria following the incubation of the implants with an inoculum of 2 × 10^3^ CFU/ml for 24 h ∗, *p* < 0.05, ∗∗, *p* < 0.01, ∗∗∗, *p* < 0.001. *n* = 3 per group for all experiments. MRSA, methicillin-resistant *Staphylococcus aureus;* PBS, phosphate buffered saline; CFU, colony forming unit.

Sr and Ag ions were released from the biofunctionalized specimens up to 28 days (Fig. 3A,B). Ion release was highest in the first 4 days followed by a gradual release profile. Sr ion release was up to 1.15 times higher (*p* < 0.01) for the PT-Sr specimens than those from the PT-AgSr group while the release of Ag ions was 1.23 times higher for the PT-Ag group than the PT-AgSr implants (*p* < 0.01).

#### 3.3.2 MIC and zone of inhibition

The MIC values for Ag^+^ and Sr^2+^ were 16 μM and 20 mM, respectively, while combining 4 μM of Ag^+^ and 2.5 mM of Sr^2+^ prevented bacterial growth altogether (Fig. 3C). Similarly, the MBC values of Ag^+^ and Sr^2+^ were, respectively, 256 μM and 80 mM while combining 128–16 μM of Ag^+^ with 5–40 mM of Sr^2+^ resulted in total absence of bacterial growth (Fig. 3D). After 24 h incubation, PT-AgSr implants demonstrated a significantly enhanced zone of inhibition (1.52 versus 1.12 cm^2^, *p < 0.05*) as compared with the specimens from the PT-Ag group, whereas no inhibition zones were detected for the NT, PT, and PT-Sr implants (Fig. 3E).

#### 3.3.3 Quantification of bactericidal activity and prevention of biofilm formation

**Fig. 4:**
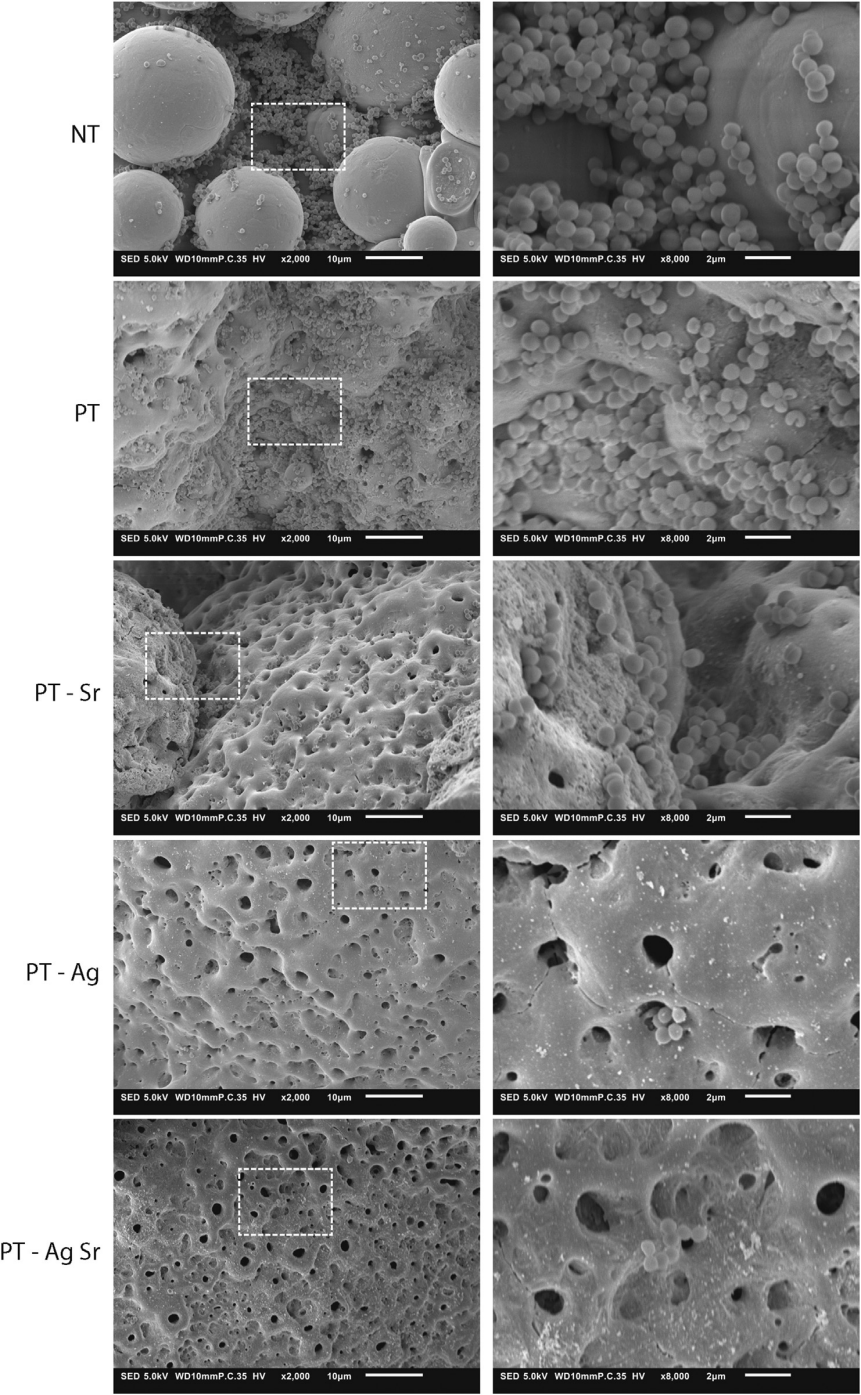
Low (2000×) and high (8000×) magnification SEM images of the MRSA USA300 bacteria and their biofilm formation on the specimens after 48 h of incubation in TSB 1% glucose. MRSA, methicillin-resistant *Staphylococcus aureus;* SEM, scanning electron microscopy; ICP-OES, inductively coupled plasma optical emission spectroscopy; TSB, tryptic soy broth.

Both PT-Ag and PT-AgSr completely prevented bacteria from adhering onto the surface after 24 h (Fig. 3F). Furthermore, PT-Ag and PT-AgSr implants eradicated all non-adherent bacteria (Fig. 3G). The NT, PT and PT-Sr implants did not prevent the growth of either adherent or non-adherent bacteria after 24 h. After 48 h, the specimens from the NT, PT and PT-Sr groups demonstrated bacterial adhesion on a substantial part of their surface area, whereas PT-Ag and PT-AgSr demonstrated almost no attached bacteria, save for a few found after substantial effort (Fig. 4). On the surface of the NT implants, these clusters of bacteria had grown into multiple layers of bacterial cells. After 48 h, no instances of stacked bacterial clusters were found on the surfaces of the PT-Ag and PT-AgSr implants.

#### 3.3.4 Ex vivo antibacterial activity

**Fig. 5:**
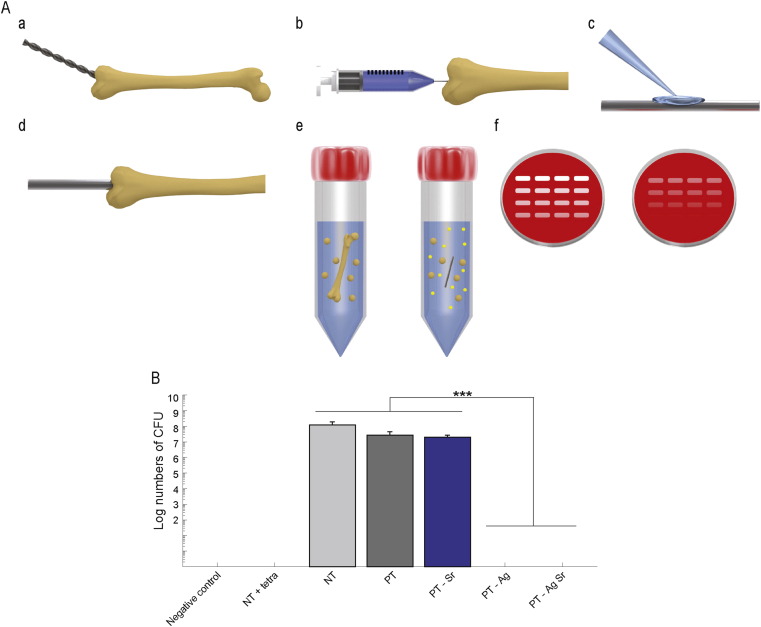
Bactericidal activity of implants in an *ex vivo* femoral mouse model against MRSA USA300. (A) (a) A hole was drilled through the epicondyle of the femur starting under an angle of 45° and lowering to the longitudinal axis of the femur. (b) Subsequently bone marrow was removed and (c) implants were inoculated with 2 × 10^2^ CFU before (d) implantation. (e) After 24 h incubation, the femora were homogenized and (f) the number of CFU was determined. (B) Number of CFU in murine femurs after 24 h incubation *ex vivo*. To confirm sterilization, a femur without implant and bacterial inoculum was processed and analyzed (negative control). To validate the model, 2 μl of tetracycline was injected into the femoral cavity prior to implantation (NT + tetra). *n* = 3, ∗∗∗, *p* < 0.001. MRSA, methicillin-resistant *Staphylococcus aureus;* CFU, colony forming unit.

The antibacterial activity was determined *ex vivo* using an intraosseous infection model consisting of murine femora (Fig. 5A). The specimens from the PT-Ag and PT-AgSr groups fully eradicated the bacterial inoculum while those from the NT, PT, and PT-Sr did not prevent bacterial growth *ex vivo* (Fig. 5B).

### 3.4 Osteogenic activity of MC3T3-E1 cells on biofunctionalized implants

**Fig. 6:**
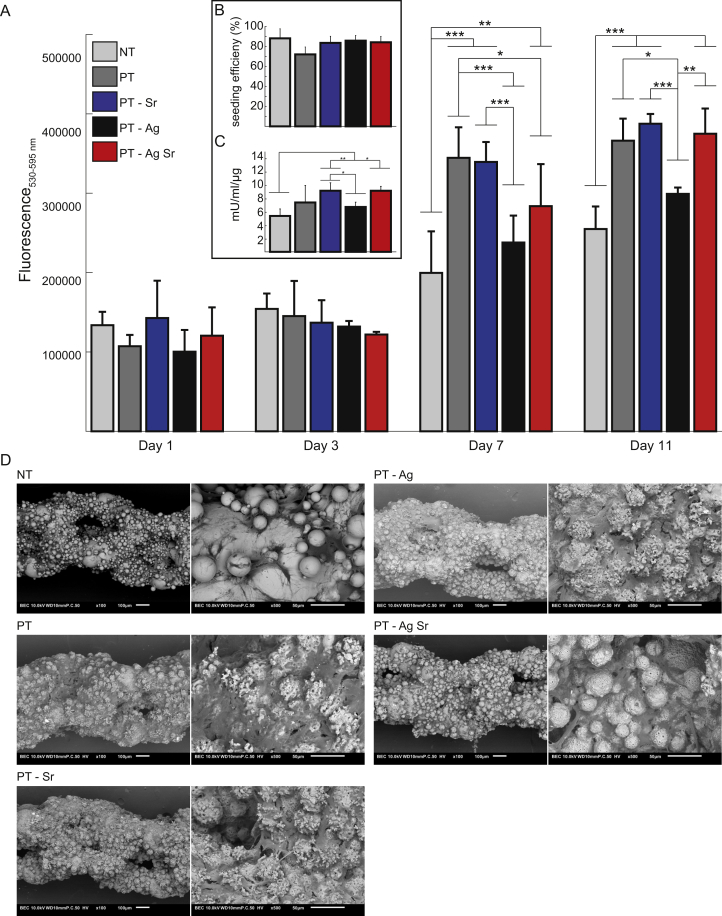
The osteogenic activity of MC3T3-E1 cells cultured on NT and biofunctionalized implants (*n* = 4 per group). (A) The metabolic activity of MC3T3-E1 cells determined by the Presto blue assay after 1, 3, 7 and 11 days of culture. (B) Cell seeding efficiency defined as % of cells present on the implants after cell seeding compared to the total number of seeded cells. (C) The ALP activity of MC3T3-E1 cells after 11 days of culture. (D) SEM images revealing the cell morphology and spread of MC3T3-E1 cells on the specimens after 11 days of culture (magnification: 100×and 500×). ∗, *p* < 0.05, ∗∗, *p* < 0.01, ∗∗∗, *p* < 0.001. ALP, alkaline phosphatase; SEM, scanning electron microscopy.

After 1 and 3 days, the metabolic activity of the MC3T3-E1 cells on all specimens was similar, whereas after 7 and 11 days the metabolic activity on specimens from the PT, PT-Sr and PT-AgSr groups was significantly enhanced as compared with the NT implants (*p* < 0.001 and *p* < 0.01, respectively; Fig. 6A). In addition, after 7 days metabolic activity of the PT implants was higher than those from the PT-Ag and PT-AgSr groups (*p* < 0.001 and p < 0.05, respectively), as well as PT-Sr compared with PT-Ag (<0.001). Furthermore, after 11 days the metabolic activity of PT-Sr, PT-AgSr, and PT implants was enhanced compared with PT-Ag implants (*p* < 0.001, *p* < 0.01 and *p* < 0.05, respectively). The efficiency of the cell seeding was 83 ± 6% and did not differ significantly between experimental groups (Fig. 6B). After 11 days, the ALP activities of the specimens from the PT-Sr and PT-AgSr groups were significantly higher than the NT implants (*p* < 0.01 and *p* < 0.05, respectively), as well as PT-Sr compared with PT-Ag (*p* < 0.05; Fig. 6C). Although all surfaces demonstrated cell attachment on substantial parts of their surfaces, the surface of all biofunctionalized implants was almost fully covered by the cells (Fig. 6D). Cells demonstrated elongated morphologies and were attached onto and into the micropores. Furthermore, the cells crossed the gaps in between the 3D morphology of the implant surfaces.

## 4 Discussion

We presented an advanced prototype of a functionality-packed porous metallic biomaterial made through AM and surface biofunctionalized to stimulate its bone regeneration performance and to prevent IAIs. The results presented here clearly verified the presence of all intended functionalities and revealed a number of unique features that this biomaterial possesses. In particular, we showed that these functionality-packed porous biomaterials are extremely potent against the multi-drug resistant strain MRSA USA300 that is resistant against various antibacterial agents including erythromycin, levofloxacin, mupirocin and tetracycline [48]. In fact, we observed total eradication of planktonic and adherent bacteria both in our *in vitro* experiments, as well as in our *ex vivo* murine femoral model.

Antibacterial surfaces based on silver that exhibit strong antibacterial activities are usually extremely cytotoxic against host cells to the point that very few to no viable host cells could be found *in vitro* [49]. The biomaterials biofunctionalized with silver nanoparticles presented here, however, exhibit a combination of significantly increased osteogenic activity with unusually high levels of bactericidal behavior against a potent multidrug-resistant bacterial strain. On top of these unique multifunctional properties, we reported for the first time the synergistic antibacterial behavior of silver and strontium ions, which could be used to decrease the required concentration of silver ions by 4–32 folds. Such a huge decrease in the required concentration of silver ions (corresponding to the synergistic levels of MIC and MBC) allows for minimizing cytotoxicity against host cells while fully eradicating multidrug-resistant bacterial strains that form severe infection burdens for patients and for health-care systems worldwide.

Combined with other functionalities such as a fully interconnected porous structure, bone-mimicking mechanical properties, form-freedom allowing for the design of patient-specific implants, and highly increased surface area that amplifies the effects of biofunctionalized surfaces, the functionality-packed AM porous biomaterials presented here constitute a very promising candidate for fabrication of a new generation of orthopedic implants.

Morphological assessment of the AM porous implants confirmed that a number of design objectives that were set out to obtain the required functionalities have been achieved. The results of this study clearly show a fully interconnected porous structure with a regular, ordered topology that closely matches the design, an increased surface area, micro/nanotopographical features that are known to improve cell attachment [[50], [51], [52]], the formation of hydroxyapatite (Ca_10_(PO_4_)_5.64_(CO_3_)_0.66_(OH)_3.03_) and strontium apatite (Sr_5_(PO_4_)_3_(O_2_)_0.24_(OH)_1.52_) that stimulate bone tissue regeneration as a result of the same one-step biofunctionalization process [36,[53], [54], [55]], and continuous release of both Sr^2+^ and Ag^+^ for up to 4 weeks. This confirms the functionality-packed nature of the presented porous biomaterial.

Permanent protection against both septic and aseptic loosening requires that the release of active agents continues for several years particularly in the case of antibacterial agents. That is because bacteria may be able to reach the implant surface even years after the surgery through the blood stream or as a consequence of infection in a nearby organ [20]. Such long-term release of antibacterial agents is, first of all, not easily achieved using most biofunctionalization techniques and may not be even desired in the case of antibiotics. That is because the depletion of the reservoir of antibacterial agents will gradually result in lower concentrations being released. Long-term exposure of bacteria to sublethal doses of antibiotics is widely confirmed to result in the development of antibacterial resistance and appearance of multidrug-resistant bacteria that are not easily treated.

The biomaterials developed in the present study offer three advantages in this regard. First, immobilization of the silver nanoparticles within the firmly attached oxide layer that grows from the bulk of the biomaterial itself ensures very long-term delivery of the active agents [23,56]. Second, it is known that, as opposed to antibiotics, bacteria do not easily acquire resistance against silver ions [57]. Indeed, long-term delivery may be only advisable for the antibacterial agents against which bacteria do not easily acquire resistance such as silver ions. Finally, the synergistic behavior resulting from simultaneous release of silver and strontium ions results in an unusually strong antibacterial behavior, which is expected to be even more difficult for the bacteria to acquire resistance against.

While a beneficial osteogenic effect of silver addition to strontium-containing surfaces has been described [58], in this study, for the first time ever a synergistic antibacterial behavior between silver and strontium is reported. The underlying mechanism of this behavior is not clear, yet our MIC and MBC measurements clearly show that between 4- and 32-folds lower concentrations of silver are required to inhibit growth and kill bacteria depending on the concentration of strontium ions available in the solution. The release of strontium ions at concentrations of about 10-fold higher than silver ions may change the peri-cellular environment locally (e.g. increase in pH, osmotic pressure [59,60]) and influence molecular interactions with the cell wall, potentially favoring the ingress of silver ions, in addition to their own inhibitory effects on bacteria via inactivation of ATP synthesis and induced oxidative stress [61]. Similar types of synergistic behavior have been previously shown when silver has been combined with antibiotics (e.g. vancomycin [28]) or other inorganic materials such as zinc [62] or copper [63]. However, the synergistic behavior observed here has a major advantage over all those reported previously: strontium is not known to cause bacterial resistance such as those caused by antibiotics nor does it cause cytotoxicity at doses reported for other metallic ions [64]. Indeed, our results demonstrate an improved cell response and an osteogenic behavior which is beneficial for improving the bone tissue regeneration performance of our biomaterials.

To fully exhibit the functionality of the developed biofunctionalized AM porous implants, *in vivo* studies are to be conducted that include an active immune system which apart from preventing infection strongly affects bone regeneration [65]. Prior studies on silver-bearing biomaterials demonstrated strong antibacterial behavior *in vitro* [28,66,67] yet showed varying results *in vivo* where silver was capable to prevent bacterial adhesion [68,69] while it may also hamper the immune response to infection [29]. Meanwhile, strontium has shown to compensate for the observed cytotoxic effects of silver [70,71] and promote bone formation in critical-sized defects [72]. Furthermore, strontium reduces osteoclastogenesis and modulates the macrophage response towards enhanced bone formation [73,74].

Evidence is mounting in support of the osteogenic behavior of strontium [[75], [76], [77], [78], [79]]. Although the exact mechanisms are not yet fully understood, it is known that strontium enhances osteoblast activity and inhibits bone resorption via activation of the calcium-sensing receptor, upregulation of osteoprotegerin and downregulation of RANKL expression [80,81]. A local and sustained release of therapeutic levels of strontium in the peri-implant area can stimulate bone formation while eliminating the adverse side effects associated with a systemic treatment.

Next to its osteogenic effect, strontium may stimulate angiogenesis which is essential for osteogenesis [82]. Our results also support the osteogenic behavior of strontium including significantly increased ALP activity. This enhanced osteogenic response means that our biomaterials satisfy one of the other design objectives, required for secondary fixation of orthopedic implants.

## 5 Conclusions

In summary, we presented an AM porous biomaterial with the full range of the functionalities that are required to enhance the longevity of orthopedic implants to the point that neither septic nor aseptic loosening will occur throughout their expected service life. The AM porous biomaterials were biofunctionalized using PEO to incorporate multiple active agents (i.e. silver nanoparticles and strontium) into the micro- and nanotopographical structure that uniformly covered their entire surface. Moreover, the same single-step process also integrated hydroxyapatite into the biofunctionalized oxide layer. Our results confirm that this biomaterial satisfies all the design criteria set out and is packed with the full range of intended functionalities including a much larger surface area, a fully interconnected porous structure and most importantly a combination of strong antibacterial and osteogenic behaviors. The data resulting from both our *in vitro* experiments and *ex vivo* murine model show total eradication of both planktonic and adherent MRSA within 24 h. Furthermore, our biomaterials resulted in significantly higher level of ALP activity compared with non-biofunctionalized implants, confirming their osteogenic response. Finally, we discovered an unexpected synergistic antibacterial behavior between silver ions and strontium that is of tremendous potential use, given that it allows for simultaneously reducing the required dose of silver ions by 4–32 folds while inducing osteogenic behavior. The functionality-packed biomaterials presented here therefore have a unique potential for clinical applications and prolonging the longevity of orthopedic implants.

## Credit author statement

**I.A.J. van Hengel**: Conceptualization, Investigation, Methodology, Writing – Original Draft, Writing – Review & Editing, Visualization, Supervision; **F.S.A. Gelderman**: Investigation, Methodology; **S. Athanasiadis**: Investigation, Methodology; **M. Minneboo**: Investigation, Methodology; **H. Weinans**: Writing – Review & Editing; **A.C. Fluit**: Resources, Writing – Review & Editing; **B.C.J. van der Eerden**: Methodology, Resources, Writing – Review & Editing, Supervision; **L.E. Fratila-Apachitei**: Conceptualization, Methodology, Resources, Writing – Review & Editing, Supervision; **I. Apachitei**: Conceptualization, Methodology, Resources, Writing – Review & Editing, Supervision; **A.A. Zadpoor**: Conceptualization, Resources, Writing – Review & Editing, Supervision, Funding Acquisition.

## Declaration of competing interest

The authors declare that they have no known competing financial interests or personal relationships that could have appeared to influence the work reported in this paper.
